# The evolution of direct transcranial photobiomodulation: a history of dose, depth, and delivery

**DOI:** 10.3389/fpsyt.2026.1970464

**Published:** 2026-10-09

**Authors:** Luis De Taboada, Jackson Streeter

**Affiliations:** 1 NeuroThera, Newark, DE, United States; 2 UF Innovate | Ventures, University of Florida, Gainesville, FL, United States

**Keywords:** acute ischemic stroke, major depressive disorder, near-infrared light, neuromodulation, optical dosimetry, photobiomodulation, target engagement, transcranial

## Abstract

Direct transcranial photobiomodulation (dtPBM) delivers red or near-infrared light through the scalp and skull with the aim of affecting brain tissue. The field has produced encouraging findings but inconsistent clinical results. We reconstruct its development as a history of scientific and engineering decisions in which optical access, target depth, and dose repeatedly shaped what could be tested. The clinical lineage began with light therapy for ischemic myocardium and moved to stroke, where cadaver measurements established that near-infrared light could traverse the human scalp and skull and defined a historical design target of approximately 7.5 mW/cm^2^ at the dura. The NEST clinical program then carried a fixed 808-nm regimen into three stroke trials: NEST-1 met its primary endpoint, NEST-2 showed a nonsignificant overall trend (p = 0.094) with a published *post hoc* signal in moderate stroke (p = 0.044), and NEST-3 stopped for futility. An unpublished, exploratory NEST-2 reanalysis described larger treatment differences for superficial lesions; this observation motivates prospective testing but does not establish depth-dependent efficacy. After NEST, treatment moved to the forehead, where target geometry was more reproducible. The ELATED trials adopted a more practical LED platform at substantially lower irradiance while preserving broadly similar treatment-site radiant exposure, whereas the TRIADE R61 trial deliberately varied optical parameters and found optical-parameter-dependent, bidirectional BOLD power responses. The historical record also leaves biological mechanism unresolved: mitochondrial/redox, neurovascular, glial-inflammatory, network-mediated, and diffuse or systemic pathways remain plausible at different levels of evidence, and a distributed physiological response does not by itself identify where the effect begins. These observations support a constrained historical inference: dose, depth, and delivery site are biological variables, not merely device specifications. We distinguish focal-local, diffuse/systemic, and local-stimulation/network-mediated accounts of action and identify prospective tests that can discriminate among them. The next phase of dtPBM development should prioritize indications in which optical access, target engagement, and a disease-relevant network or biomarker can be specified prospectively rather than inferred after a clinical outcome.

## 1 Introduction

Direct transcranial photobiomodulation (dtPBM) means delivering red or near-infrared light through the scalp and skull with the intention of irradiating brain tissue for a clinical benefit. The qualifier “direct” is used here only to specify the delivery route. It does not assume that the clinically relevant effect begins in the illuminated tissue, which is one of the questions this history exposes. Intranasal, remote, and whole-body photobiomodulation are outside the scope except where they bear directly on that mechanistic question.

This is a historically structured Hypothesis and Theory article rather than a systematic review. It follows the PhotoThera/NEST development lineage because its continuity makes it possible to trace experimental decisions and their consequences across more than two decades, while introducing parallel programs when they changed the trajectory of the field (**Figure 1**). The purpose is not to identify a single mechanism retrospectively, but to use that evolution to separate questions that were often addressed at different times: how much light reaches a named intracranial target, which biological compartment responds first, and how that response relates to a disease-relevant circuit or substrate.

**Figure 1:**
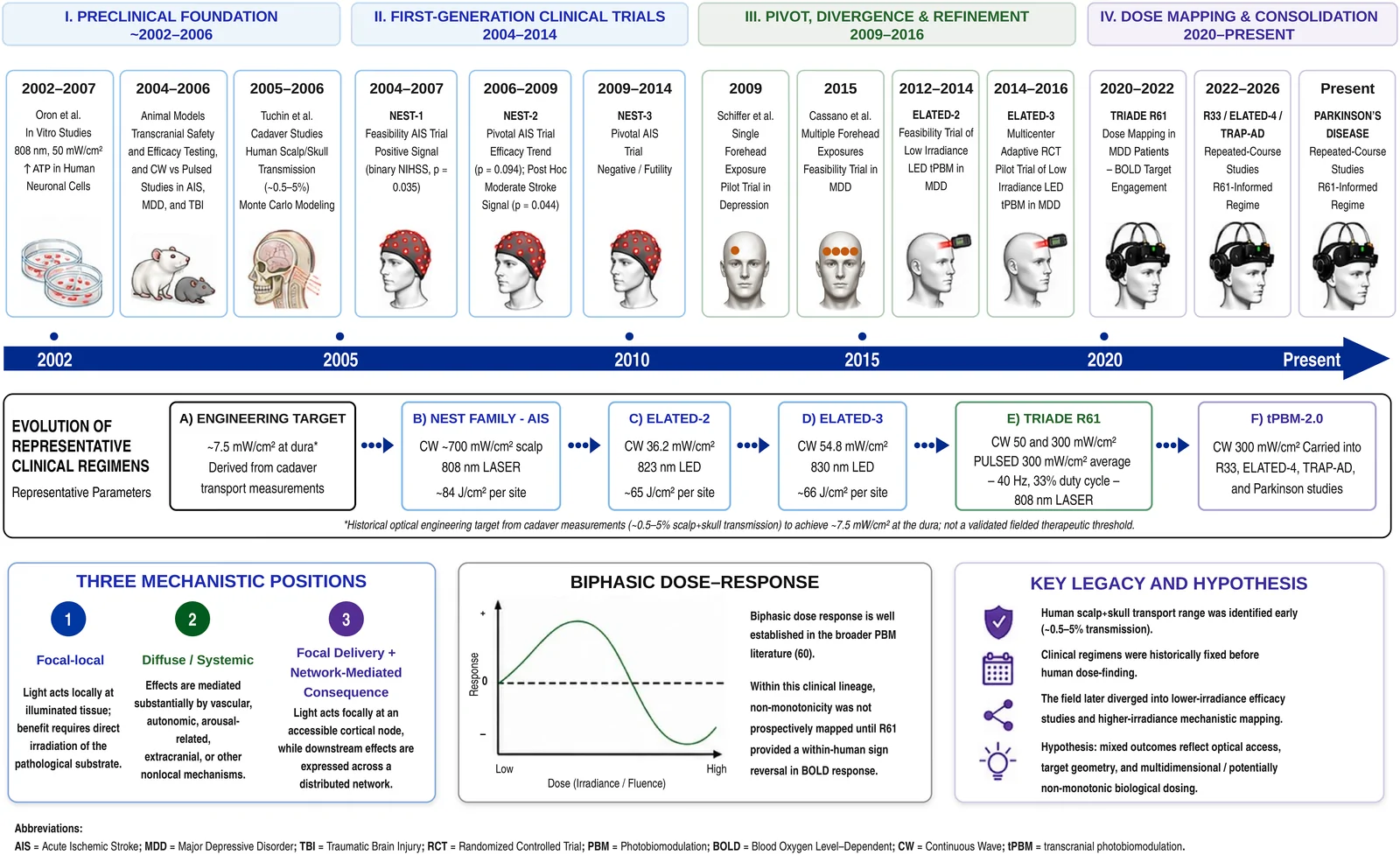
Evolution of dtPBM as a translational history of optical access, clinical-regimen development, and mechanistic refinement. The upper timeline follows preclinical work, human cadaver transport, NEST, the forehead/ELATED pivot, TRIADE R61, and subsequent tPBM-2.0 programs. The middle strip summarizes representative regimen parameters and the historical ~7.5 mW/cm^2^ dural engineering target. The lower panels summarize three mechanistic positions, the biphasic dose-response concept (60), and the hypothesis developed in this article. The biphasic curve is a generic schematic of non-monotonicity reported in the broader photobiomodulation literature (60); it is not a plot of the R61 result, and its limb ordering should not be mapped onto the R61 conditions, in which the lower-irradiance condition decreased and the moderate-irradiance CW condition increased BOLD power. Its horizontal axis denotes optical dose schematically; as set out in Section 9, irradiance and radiant exposure are not interchangeable descriptors. The proposed contribution of optical access, target geometry, and dose to mixed clinical outcomes is hypothesis-generating and is not established by the historical sequence. The ~0.5%–5% scalp-plus-skull transmission range and ~7.5 mW/cm^2^ dural value are historical engineering quantities, not validated therapeutic thresholds.

The lineage did not originate photobiomodulation itself. Endre Mester’s late-1960s laser-biostimulation observations established the broader low-level-light tradition. In neural tissue, Mochizuki-Oda, Kataoka and colleagues reported 830-nm-associated changes in ATP and ADP in rat brain in 2002, and Wong-Riley, Eells, Whelan, Chance and colleagues later linked red/near-infrared exposure to cytochrome-c oxidase and neuronal bioenergetics (1–3).

The clinical history discussed here began not in the brain, but in the heart.

## 2 From the heart to the brain

Uri Oron and colleagues showed in rats and dogs that low-energy laser irradiation could reduce infarct size and scar formation after experimental myocardial infarction (4, 5). Streeter, De Taboada and Oron summarized the emerging mitochondrial and cytoprotective rationale in 2004 (6).

Translation to the human heart met a physical barrier. Externally applied photons would have to traverse skin, fat, muscle, ribs, and lung before reaching myocardium. The thoracic path was long, highly scattering, and strongly dependent on body habitus. A fixed external dose therefore could not be expected to generate a reproducible myocardial exposure.

Stroke offered a shorter and more predictable optical path. Acute ischemic stroke also shared the biological features that made myocardial infarction attractive: abrupt loss of blood flow, mitochondrial dysfunction, oxidative stress, inflammation, and potentially salvageable tissue. The question became whether enough near-infrared light could cross scalp and skull to reach cortex.

Working with Valery Tuchin’s Institute of Optics and Biophotonics at Saratov State University, Vladislav Lychagov, Evgeny Savchenko and colleagues measured 810-nm transmission through twenty human cadaver heads at five anatomical regions, with shaved scalp in place and with skull alone (7, 8). Scalp-plus-skull transmittance ranged from approximately 0.5% to 5%, while skull-alone transmittance ranged from 1% to 16%, falling exponentially with thickness. At an incident irradiance of 68 mW/cm^2^, the reported transmitted irradiances ranged from 0.34 to 3.4 mW/cm^2^ with scalp and 0.68 to 9.0 mW/cm^2^ without it. The scalp, although thinner than the skull, contributed substantial attenuation.

Thermometry and modeling were performed in the same development program. Skin-surface temperature rose by about 4 °C over a four-minute exposure and the outer skull by about 2 °C, with little change at the inner skull surface; because cadaver tissue is unperfused, those measurements are conservative with respect to *in vivo* heat clearance (7, 8). A matched Monte Carlo model reproduced the observed decay across layered scalp and skull and published the optical properties used in the simulation (9).

From these studies came a brain-level engineering target of approximately 7.5 mW/cm^2^ at the dura (7–9). Its provenance was translational rather than neurological: the myocardial program had shown its largest effect near 6 mW/cm^2^ at the epicardium. The 7.5-mW/cm^2^ value was therefore a design target carried between organs, not a validated minimum effective cortical dose.

By 2006 the program had a measured human transport range, a target at a named anatomical plane, and a thermal bound. What it did not have was a way to measure target exposure in individual patients or a human dose-finding study.

## 3 The PhotoThera preclinical program

Between roughly 2002 and 2007, under Jackson Streeter’s direction at PhotoThera, the program assembled a translational package spanning optical characterization, device engineering, transcranial efficacy, a second-species embolic model, dose-escalation safety, toxicology, and regulatory development. Many efficacy experiments delivered light through intact scalp and skull rather than exposed cortex, so optical attenuation was built into the preclinical testing. Several hundred animals were studied before the first patient was treated.

De Taboada and colleagues induced stroke in 169 rats and randomized them to no laser or to transcranial 808-nm irradiation delivered ipsilateral, contralateral, or bilateral to the lesion (10). All irradiated groups improved significantly at 14, 21, and 28 days. Oron and colleagues, using the same model, found that treatment initiated 24 hours after stroke reduced long-term neurological deficit and associated the effect with neurogenesis rather than acute salvage (11). The contralateral result raised an early spatial inference: benefit did not require the source to sit over the injured hemisphere. However, because a rat brain is small and the skull is thin, this does not establish a nonlocal mechanism and cannot validate human-scale spatial targeting. Later interspecies measurements confirmed large differences in calvarial transmission (12).

Lapchak, Wei and Zivin extended the program to a rabbit small-clot embolic model and reported improved clinical rating scores after transcranial near-infrared treatment (13). An extended-window study compared continuous and pulsed delivery and again reported benefit (14), and a later study found increased cortical ATP after treatment (15). The rabbit work supplied efficacy in a second species and introduced temporal mode into the translational evidence base.

Ilic and colleagues then tested 808-nm transcranial irradiation across 7.5, 75, and 750 mW/cm^2^ with different pulse frequencies and session counts (16). Only the 750-mW/cm^2^ continuous-wave (CW) condition produced neurological deficit and histopathological injury, attributed to heating. Pulsed delivery at the same average condition produced substantially less heating and no injury. The study established a practical upper safety bound, but it also showed that temporal structure could change the biological and thermal consequence of CW exposures at nominally similar average irradiance.

Traumatic brain injury studies made that point more directly. Oron and colleagues first reported improved long-term outcomes when 808-nm treatment was delivered 4, 6, or 8 hours after closed-head injury (17), then compared CW with 100-Hz and 600-Hz pulsed delivery; full neurological recovery at 56 days was highest in the 100-Hz group (18). Ando and colleagues, in a different model with matched average irradiance and fluence, found 10 Hz stronger than CW or 100 Hz (19). The result was not a universal optimal frequency. It was evidence that temporal structure mattered even when total energy was similar, a conclusion also emphasized in a 2010 review of pulsing in low-level-light therapy (20).

A chronic amyloid model separated additional components of dose. In amyloid-β protein precursor transgenic mice, six months of transcranial 808-nm treatment reduced plaque burden and inflammatory markers and increased ATP and mitochondrial function across CW and pulsed conditions (21). All pulsed formats used 100-Hz, 2-ms pulses, but one matched CW peak power while delivering one fifth the average power and energy, whereas another matched CW average power and fluence while increasing peak power fivefold. Different outcomes under those comparisons show why “dose” cannot safely be reduced to J/cm^2^ alone. R61 later used 40-Hz pulsing, a configuration distinct from the 10/100/600-Hz preclinical paradigms, contemporaneous with interest in 40-Hz sensory entrainment following Iaccarino et al. (22).

A parallel thread in Juanita Anders’s laboratory extended 810-nm treatment into spinal-cord injury and a pulsed chronic-stress model of depression (23–25). Repeated treatment was also examined for long-term safety (26), while imaging studies of the period documented persistence of viable hypoxic tissue for roughly 24–48 hours in some stroke patients (27, 28). The acute efficacy models, however, did not establish whether repeating treatment within that window improved outcome.

The preclinical program therefore established transcranially delivered biological effects across multiple models and a broad thermal safety envelope. It did not establish a minimum effective intracranial exposure, a human dose-response relation, an optimal treatment schedule, or the anatomical locus at which the effect begins. Those unresolved quantities became central in the clinical record.

## 4 The NEST stroke trials

From 2004 to 2014, the NeuroThera Effectiveness and Safety Trial (NEST) program treated more than 1,300 patients while carrying essentially the same optical regimen through three successively larger trials.

The NeuroThera Laser System delivered 808-nm CW light to approximately twenty predefined sites across the closely shaved cranial vault, two minutes per site, at about 700 mW/cm^2^ at the scalp (approximately 84 J/cm^2^ per site). Published descriptions estimated cortical exposure near 10 mW/cm^2^ and 1.2 J/cm^2^ per site (29, 30); these were modeled estimates, not patient-level measurements. The design distributed treatment across the vault because lesion location could not be targeted reliably and used active cooling to manage scalp heating.

NEST-1 was the first human trial of transcranial laser therapy. Lampl, Zivin, Fisher, Oron and the NEST-1 investigators randomized 120 patients with acute ischemic stroke 2:1 to active treatment or sham within 24 hours of onset (29). The first-in-human treatment was performed by Cesar Caparo in Arequipa, Perú. The prospectively defined primary endpoint — complete recovery (NIHSS 0–1) or improvement of at least 9 points at 90 days — was met in 70% of treated patients versus 51% of controls (p = 0.035 after stratification by baseline severity and time to treatment). Secondary analyses were concordant, including mRS 0–2 in 59% versus 44% (p = 0.034) and a positive mRS shift analysis (p = 0.020). Serious adverse events and mortality rates did not differ significantly. Following the positive NEST-1 result, the single-session, fixed-irradiance, whole-vault CW regimen advanced into pivotal development without an intervening human dose-optimization stage.

NEST-2 randomized 660 patients; 658 contributed to the intention-to-treat efficacy analysis (30). The primary endpoint, 90-day mRS 0–2, was not met: 36.3% of treated patients versus 30.9% of sham patients achieved the endpoint (p = 0.094; adjusted OR 1.38, 95% CI 0.95–2.00). A published *post hoc* analysis of patients with baseline NIHSS 7–15 found 51.6% versus 41.7% achieving mRS 0–2 (p = 0.044). A later pooled analysis of NEST-1 and NEST-2 (n = 778) also favored treatment (p = 0.003; OR 1.67, 95% CI 1.19–2.35) (31). These findings were encouraging but could not identify a dose mechanism because the optical regimen was fixed.

A later retrospective analysis by the present authors asked whether outcome varied with lesion depth. Digitized NEST-2 CT scans were classified as superficial (approximately within 25 mm of the cortical surface), mixed-depth, or deep. Among 542 participants whose lesion depth could be classified, active/sham group sizes were 104/97, 123/126, and 50/42, respectively. The treatment difference in 90-day mRS ≤2 was +10.9 percentage points for superficial lesions (49.0% versus 38.1%; Wald 95% CI −2.7 to +24.5 points), −1.9 points for mixed-depth lesions, and +0.3 points for deep lesions. Restricting the analysis to 404 participants with acute lesions and baseline NIHSS ≤17 yielded a +16.2-point difference for superficial lesions (57.5% of 87 versus 41.3% of 80; two-tailed Fisher exact nominal p = 0.044; Wald 95% CI +1.2 to +31.2 points), compared with +3.0 points for mixed-depth and −1.0 point for deep lesions. No treatment-by-depth interaction test was performed. The analysis was *post hoc*, unpublished, not prespecified, and unadjusted for multiplicity. It is reported here as hypothesis-generating because lesion depth is directly relevant to optical accessibility. The reported subgroup results do not by themselves establish a treatment-by-depth interaction or a causal effect of lesion depth on treatment response.

NEST-3 tested the severity signal prospectively by restricting enrollment to NIHSS 7–17 and excluding strokes confined to the brainstem or cerebellum, small deep infarctions, and massive hemispheric strokes (32, 33). Planned enrollment was 1,000. The Data Monitoring Committee recommended termination after a futility analysis of 566 completed participants, and the null result remained across all 630 randomized participants (33).

NEST-3 therefore weakened the published severity-selection explanation for NEST-2. Although it excluded small deep infarctions and strokes confined to the brainstem or cerebellum, it did not measure patient-level target exposure or use optical accessibility as an entry variable. More importantly for this history, it changed the population but not the intervention: wavelength, scalp irradiance, temporal mode, treatment geometry, and session count remained essentially those selected before NEST-1.

The stroke program established that this fixed regimen was feasible and did not improve outcome in the definitive trial. Because individual cortical exposure, target engagement, and dose-response were never measured, the program could not determine whether another optical condition would have produced a different biological response.

## 5 From the entire cranium to the forehead

After NEST, treatment moved increasingly from the hair-bearing vault to the forehead. The change improved both ends of the optical problem: the forehead offered a more standardized, relatively hair-sparse coupling surface, and depression studies allowed the target to be specified before treatment rather than determined by an unpredictable lesion.

Schiffer, Johnston, Ravichandran, Webb, Hamblin and colleagues reported psychological benefit after a single near-infrared forehead exposure in ten patients with depression and anxiety in 2009 (34). Cassano and colleagues then made the direct bridge back to the NEST program by applying the same NeuroThera system to four forehead sites at the NEST parameters — 808 nm CW, approximately 700 mW/cm^2^, 84 J/cm^2^ per site, two minutes per site — twice weekly for three weeks (35). Only four participants completed the crossover study, so it could not establish efficacy. Its historical importance is that the NEST source and exposure were reused at a different optical window and with repeated sessions.

Parallel programs were already widening the field. Naeser and colleagues developed repeated transcranial red/near-infrared LED treatment for chronic traumatic brain injury (36, 37), while Barrett and Gonzalez-Lima established controlled human 1064-nm transcranial infrared stimulation in 2013 (38). These programs were not descendants of the NEST dosing paradigm and should not be treated as interchangeable evidence because source, wavelength, coupling, schedule, and target differed.

PhotoThera closed in 2012 following the NEST-3 result, ending the company but not the device-development thread. The program continued through LiteCure and later NeuroThera into ELATED, TRIADE, TRAP-AD, and subsequent tPBM-2.0 studies (39–42).

The ELATED studies used a more practical LED platform at substantially lower irradiance. LED arrays replaced the actively cooled laser platform, reducing scalp irradiance from approximately 700 mW/cm^2^ in NEST/Cassano to 36.2 mW/cm^2^ in ELATED-2 and 54.8 mW/cm^2^ in ELATED-3, while treatment duration was lengthened so that treatment-site radiant exposure remained in the same general range (39, 40). ELATED-2 (n = 21) produced an uncertain efficacy signal; ELATED-3 (n = 49) did not meet its primary endpoint.

This transition matters because it operationally treated irradiance and time as partly substitutable. The preclinical record argues that they need not be. Preserving J/cm^2^ while changing irradiance, peak power, or temporal structure does not guarantee biological equivalence.

## 6 TRIADE: testing optical dose

TRIADE R61 changed the development logic by making optical parameters independent variables within a single human study. The NIMH R61/R33 program, led by Iosifescu and Cassano, compared sham with three 808-nm conditions delivered bilaterally at F3/F4 in 31 participants with major depressive disorder: 50 mW/cm^2^ CW, approximately 300 mW/cm^2^ CW, and a pulsed condition at approximately 300 mW/cm^2^ average irradiance, 40 Hz, 33% duty cycle, designated low, medium, and high dose in the primary report (41, 43).

BOLD was recorded before, during, and after stimulation, and the reported comparison was the change in BOLD power during stimulation relative to sham. Relative to sham, the 50-mW/cm^2^ condition significantly decreased BOLD power and the approximately 300-mW/cm^2^ CW condition significantly increased it, while the pulsed condition produced no significant mean change in the prespecified regions; direct contrasts between the active conditions were not reported, so the difference between them is established by the omnibus dose-by-time interaction rather than by a pairwise estimate. An exploratory whole-cortex analysis did report a small decrease under the pulsed condition. The CW pattern held in the middle frontal gyrus, in the directly irradiated prefrontal cortex, and across the cortex as a whole, so the response extended beyond the illuminated tissue. Single administrations did not change depression severity relative to sham at any condition (43). The trial therefore establishes acute, parameter-dependent physiological engagement, not clinical benefit.

Conference reports preceded the peer-reviewed primary analysis (44, 45), which provides the basis for the interpretation here. Ancillary analyses were consistently null with respect to dose: ¹H-MRS thermometry found no significant brain-temperature elevation (46), a neurometabolite analysis found no active-dose-specific metabolite effect (47), and a safety analysis found no significant dose association with adverse events or vital-sign changes (48). The pulsed condition delivered the greatest session radiant exposure yet did not reproduce the approximately 300-mW/cm^2^ CW response. Total session energy is therefore an inadequate scalar description of biological dose.

The constrained result is that changing the optical parameters changed the measured acute physiological response. The BOLD finding identifies parameter-sensitive engagement rather than the cellular mechanism that produced it, although the accompanying thermometry makes a simple thermal explanation less likely (46).

R61 also changed how parameters were propagated. Its approximately 300-mW/cm^2^ CW condition became the nominal design point for the tPBM-2.0 platform used in the subsequent R33, ELATED-4, TRAP-AD, and Parkinson programs. Unlike the NEST regimen, this condition followed a human parameter-mapping study, although R61 identified parameter-sensitive physiology rather than a validated therapeutic optimum.

For most of this history, inadequate delivery remained compatible with a negative clinical result because target exposure was not measured and optical dose was not varied. That possibility justified further investigation but did not validate the dosing hypothesis; an explanation that accommodates every outcome has not been discriminatively tested. R61 changed that logic by varying optical parameters within the same participants, creating the first direct opportunity in this lineage for the dosing account to be contradicted.

## 7 Where does the effect begin? Competing models and falsifiable predictions

R61 also sharpened a second unresolved question: locality. Bilateral frontal illumination produced a spatially distributed BOLD response, including regions outside the illuminated prefrontal cortex (43). Spatial extent alone does not identify the mechanism. At least three positions remain compatible with the record.

First, a focal-local model holds that light acts at the illuminated tissue and clinical benefit requires sufficient direct irradiation of the pathological substrate. This was the implicit logic of the stroke program.

Second, a diffuse or systemic model holds that a substantial part of the response is mediated by vascular, autonomic, arousal-related, extracranial, or other pathways that do not require local cortical photochemistry. Under this model, changing the cortical montage need not reorganize the response in a way predicted by brain connectivity.

Third, a local-stimulation/network-mediated model holds that photons act locally at an accessible cortical node, while downstream effects propagate through a distributed circuit. The diseased tissue itself need not receive a therapeutic photon dose. This model predicts distal effects, but it also predicts that the pattern of those effects should depend on which cortical node is illuminated.

The distributed R61 response is compatible with the third position but does not distinguish it from the second. A discriminating human experiment would compare anatomically distinct cortical montages under matched optical regimens, accounting for site-specific target exposure and extracranial or sensory effects, and test whether downstream responses differ as predicted by connectivity. No published human study in this lineage has performed that contrast.

Distinguishing the first from the third position requires a different test. Both posit local photon action at cortex; they differ on whether the pathological substrate itself must be irradiated. Within one indication and at matched optical parameters, benefit should therefore be tested prospectively against prespecified cortical depth or modeled exposure of the pathological tissue. The exploratory NEST-2 depth reanalysis motivates this question but is not equivalent to a prospective controlled test. A prospective focal-lesion study relating modeled target exposure to outcome, with prespecified adjustment for lesion characteristics and baseline severity, would test this prediction.

The rodent contralateral result cannot settle the issue because illumination is much less localized in a small brain beneath a thin skull (10, 12). Human engagement studies provide a separate chronology: Barrett and Gonzalez-Lima established controlled 1064-nm stimulation in 2013 (38), and Wang, Tian, Gonzalez-Lima, Liu and colleagues later measured increased oxidized cytochrome-c oxidase with hemodynamic changes using broadband near-infrared spectroscopy (49). R61 returned a prespecified physiological engagement measure to the 808-nm lineage (43). These studies show that human brain physiology can respond during transcranial illumination, but they do not yet show which engagement signal mediates durable clinical benefit.

This history motivates the hypothesis that variation in optical dose and target exposure may contribute to differences in biological engagement and clinical outcome. Where dtPBM produces clinical benefit, local initiation at an accessible cortical node followed by network propagation is one possible mechanism. Neither proposition is established by the historical record; both require prospective testing. A local-stimulation/network-mediated account would be weakened if matched stimulation of anatomically distinct cortical nodes produced the same distributed physiological pattern despite different network connectivity. A focal-local account would be weakened if, in a prospective focal-lesion study spanning a meaningful exposure range, engagement or clinical effect showed no systematic relation to prespecified modeled exposure of the pathological substrate. A general optical-dose account would be weakened if deliberate variation of the relevant dose components produced no reproducible change in an independent engagement measure. These are prospective tests; the historical record does not yet decide among them.

## 8 Alzheimer’s and Parkinson’s disease as mechanistic tests

Alzheimer’s and Parkinson’s disease are useful because their pathological anatomy differs from both focal stroke and prefrontal depression.

Alzheimer’s disease is an intermediate case. Pathology is distributed across cortical and medial temporal networks; frontal cortex is optically more accessible than deeper medial temporal structures. Frontal dtPBM is therefore most coherently framed as cortical or network stimulation rather than direct irradiation of all pathological tissue. Gaggi and colleagues reported increased intrinsic brain activity within irradiated regions in early Alzheimer’s disease (50), while the TRAP-AD protocol incorporates cognition, resting-state fMRI, ³¹P-MRS bioenergetics, and tau PET (42). The design is important because engagement and disease biomarkers are measured rather than inferred from symptoms alone.

Parkinson’s disease is the limiting case for a focal-local account. The substantia nigra lies several centimeters beneath the scalp, at a depth where conventional non-invasive frontal near-infrared illumination is expected to deliver only a very small fraction of incident optical energy, consistent with measured transcranial attenuation (51–53). A frontal transcranial Parkinson intervention therefore cannot reasonably depend on direct nigral illumination; it must invoke an accessible cortical node and its networks, or a diffuse mechanism.

The wider Parkinson literature illustrates both alternatives. Darlot, Moro, El Massri, Johnstone, Mitrofanis, Benabid and colleagues delivered 670-nm light intracranially near the substantia nigra in MPTP-treated macaques and reported clinical and dopaminergic neuroprotection, with a later higher-dose study extending the work (54, 55) and preliminary human intracranial investigation subsequently reported (56). In the opposite direction, Johnstone, El Massri, Moro, Stone, Mitrofanis and colleagues found that near-infrared irradiation delivered away from the head could protect nigral neurons in a mouse parkinsonism model, with later abdominal and leg irradiation extending that line (57, 58).

A randomized six-month study of aerobic exercise, 808-nm PBM, their combination, or control in Parkinson’s disease reported the clearest effects for exercise, selected posturography findings for PBM alone, and no additive exercise-PBM effect (59). The study was not a sham-controlled blinded PBM efficacy trial, so the PBM findings are exploratory.

Current frontal tPBM-2.0 Parkinson programs are mechanistically informative because any benefit attributable to PBM would be more plausibly explained by accessible cortical, network-mediated, or systemic pathways than by direct nigral irradiation. Parkinson’s disease therefore converts an ambiguity in the broader field into a testable mechanistic commitment.

## 9 Discussion: what the history teaches

Three conclusions emerge from this history.

First, optical geometry repeatedly constrained development. The move from myocardium to brain shortened the path and removed lung. The later move from the whole vault to the forehead improved coupling and made the target reproducible. In stroke, a whole-head montage was a rational response to an unknown lesion location, but it also distributed a fixed per-site exposure across hair-bearing scalp toward targets of variable depth. In depression, target location could be specified before treatment. Route and target geometry were therefore part of the biological experiment.

Second, dose is multidimensional. Device output, scalp irradiance, target-plane irradiance, peak power, average power, treatment duration, duty cycle, frequency, and radiant exposure describe different aspects of the intervention. Preclinical comparisons showed that matched fluence could produce different outcomes when temporal structure or peak power changed (18–21). R61 supplied the corresponding human observation: the 50- and approximately 300-mW/cm^2^ CW conditions changed BOLD power in opposite directions, while the highest-energy pulsed condition did not reproduce the medium-CW response (43). Biphasic dose-response behavior is well established in the broader photobiomodulation literature (60); R61 is notable because the two CW conditions changed BOLD power in opposite directions relative to sham within the same participants — a sign reversal in this clinical lineage, though one supported by the omnibus interaction rather than by direct active-condition contrasts.

Third, target exposure still needs to be measured. Later cadaver studies by Tedford et al. and Jagdeo et al. confirmed measurable transcranial penetration but also reinforced that transmission depends on anatomy, preparation, wavelength, source geometry, and measurement plane rather than a universal percentage (51, 52). The historical 0.5%–5% transmission range should therefore be used as an engineering reference, not as a patient-level dosimeter. More recent measurements using different devices and tissue preparations have reported substantially lower transcranial transmission, further emphasizing that penetration cannot be represented by a universal percentage and must be interpreted in relation to wavelength, source geometry, tissue preparation, and measurement plane (53).

The original 7.5-mW/cm^2^ dural target remains useful in exactly that limited sense. Applying the 0.5%–5% cadaver transmission range arithmetically, 7.5 mW/cm^2^ at the dura corresponds to roughly 150–1500 mW/cm^2^ at the scalp (7, 8). **Table 1** places representative human conditions against that historical band. The comparison mainly separates very-low-irradiance conditions from the rest: ELATED-2 and ELATED-3 remain below 7.5 mW/cm^2^ even at 5% transmission; the R61 approximately 300-mW/cm^2^ CW condition overlaps the band in higher-transmission specimens; and NEST at approximately 700 mW/cm^2^ also overlaps it despite a negative phase III trial.

**Table 1:** Representative human conditions compared with the historical 7.5-mW/cm^2^ dural engineering target.

Condition	Scalp irradiance	Arithmetic range at historical 0.5%–5% transmission	Reported result
NEST-1/2/3	~700 mW/cm^2^	3.5–35 mW/cm^2^	NEST-1 positive primary endpoint; NEST-2 overall p = 0.094 and *post hoc* NIHSS 7–15 p = 0.044; NEST-3 futility
Cassano 2015	~700 mW/cm^2^	3.5–35 mW/cm^2^	Proof-of-concept signal; 4 completers
ELATED-2	36.2 mW/cm^2^	0.18–1.81 mW/cm^2^	Uncertain efficacy signal
ELATED-3	54.8 mW/cm^2^	0.27–2.74 mW/cm^2^	Primary endpoint null
TRIADE R61 CW-50	50 mW/cm^2^	0.25–2.5 mW/cm^2^	BOLD power decreased
TRIADE R61 CW-300	~300 mW/cm^2^	1.5–15 mW/cm^2^	BOLD power increased
TRIADE R61 pulsed	~300 mW/cm^2^ average	1.5–15 mW/cm^2^ (average-irradiance arithmetic)	No significant change in prespecified BOLD ROIs; highest session radiant exposure

Arithmetic ranges are products of scalp irradiance and the historical 0.5%–5% cadaver scalp-plus-skull transmission range. They are not patient-specific dose estimates or therapeutic thresholds. Cross-device comparison is coarse because wavelength, source geometry, coupling, anatomy, temporal mode, and measurement plane differ; representative radiant exposures are reported in the text.

That last point limits the inference. Matching the historical target is neither sufficient for clinical efficacy nor evidence that 7.5 mW/cm^2^ is a threshold. The planes differ, the transmission band is wide, gray-matter exposure is lower than dural exposure, and temporal structure changes biological response. The value of the retrodiction is narrower: a target exposure was specified early, later device generations moved to substantially lower scalp irradiance, and human parameter mapping subsequently returned to a higher-irradiance condition. That pattern is worth testing prospectively rather than treating as proof.

Taken together, the historical sequence is not best read as a contest between “PBM works” and “PBM does not work.” It is a sequence in which physical access, target geometry, biological dose, and mechanism were addressed at different times and with different tools. The remaining questions therefore concern not only how much light reaches the head, but which biological process is engaged and whether the trial design can distinguish among competing explanations.

### 9.1 Biological mechanisms: distinct levels of explanation

The historical record cautions against treating “mechanism” as a single question. At least three levels should be separated: the molecular target that absorbs or responds to light, the cellular or tissue compartment in which an initial response occurs, and the systems pathway through which that response becomes clinically relevant. Red and near-infrared PBM has substantial experimental support for mitochondrial and redox effects, with cytochrome-c oxidase proposed as one of the candidate photoacceptors and downstream changes in membrane potential, ATP production, nitric-oxide/redox signaling, and transcriptional programs; photoresponsive ion-channel and calcium signaling are additional candidates (2, 3, 15, 49, 61). In neural models, these proximal effects have been associated with antioxidant, anti-inflammatory, neurotrophic, synaptic, and neurogenic responses (11, 21, 23–25, 61). Human tPBM studies have also demonstrated coupled changes in oxidized cytochrome-c oxidase and hemodynamics and, more recently, optical-parameter-sensitive BOLD power (43, 49). These observations support biological engagement but do not establish neuronal mitochondria as the sole or dominant mediator of clinical effects.

Vascular and glial compartments provide complementary or alternative routes. PBM can influence cerebral perfusion and neurovascular coupling, and BOLD itself is a hemodynamic signal; a distributed BOLD response therefore cannot be equated with distributed neuronal photochemistry (43, 49, 62). Preclinical work also supports modulation of inflammatory/glial responses and, in selected models, meningeal lymphatic drainage and clearance. The lymphatic evidence is mechanistically interesting because dural lymphatic vessels are relatively superficial, but it remains predominantly preclinical and protocol-dependent: studies at 1267 nm and, more recently, 808 nm in mice should not be generalized directly to human 808-nm dtPBM (63, 64). Remote-PBM effects in parkinsonism models likewise keep systemic routes biologically plausible (57, 58). Thus, the three spatial models above do not compete with these cellular mechanisms. A local cortical mitochondrial or vascular event could propagate through a network; a dural, vascular, or systemic event could produce distributed changes without local cortical initiation; and more than one pathway may operate. Current human evidence is insufficient to rank these as mutually exclusive primary mechanisms.

### 9.2 When a trial is a weak test of direct focal dtPBM

The history also suggests when a negative trial is a weak mechanistic test of direct focal action. Four conditions are particularly problematic: the pathological substrate lies well below the optically accessible cortex while the hypothesis assumes direct irradiation; scalp output is specified without a credible estimate of target exposure; radiant exposure is treated as equivalent despite material changes in irradiance, peak power, temporal mode, or coupling; or the montage is not linked to an accessible disease-relevant cortical node or network. These conditions should not be labeled universally invalid, because a network-mediated or diffuse mechanism could still operate. Rather, they make the experiment non-discriminating: a null result cannot distinguish absence of a therapeutic mechanism from failure to engage the hypothesized target, while a positive result may require a mechanism other than direct irradiation of deep pathology. A negative clinical result nevertheless remains evidence against efficacy of the tested regimen in the studied population.

### 9.3 Where the historical arc points next

Indication selection should therefore be driven less by disease label than by whether the mechanistic chain can be measured. The most informative development settings combine reproducible optical access, a prespecified superficial cortical target or network node, an independent target-engagement readout, and a disease-relevant physiological or biomarker endpoint. Major depressive disorder and related network-defined neuropsychiatric disorders fit this logic because prefrontal targets can be specified before treatment, repeated dosing is practical, and engagement can be assessed with imaging, spectroscopy, electrophysiology, or optical methods. Superficial cortical lesions could provide a direct test of exposure dependence if depth or modeled target exposure is prespecified rather than examined retrospectively. Early Alzheimer’s disease is attractive as a mechanistic-development setting because cortical engagement, network physiology, bioenergetics, and disease biomarkers can be measured, but frontal dtPBM should not be framed as direct irradiation of all deeper medial-temporal pathology. Parkinson’s disease is informative in the opposite way: any benefit attributable to frontal PBM would warrant investigation of cortical, network-mediated, or systemic pathways rather than assumption of direct nigral irradiation. The historical record therefore does not identify a single “best” disease; it favors indications in which competing mechanisms can be prospectively discriminated.

## 10 Limitations

This article is a hypothesis-generating historical analysis, not a systematic review or meta-analysis. It emphasizes one translational lineage in which both authors participated, creating unavoidable risks of selection and hindsight bias. Cross-study comparisons also combine different sources, wavelengths, coupling methods, anatomical targets, schedules, and outcome measures.

The 7.5-mW/cm^2^ dural value is a historical engineering target rather than a validated therapeutic threshold. The NEST-2 depth analysis is retrospective, unpublished, not prespecified, and unadjusted for multiplicity; its subgroup results do not establish a treatment-by-depth interaction. R61 BOLD power is an indirect hemodynamic engagement measure and does not establish local photochemistry, mechanism, or clinical efficacy. The mechanistic discussion integrates evidence obtained with different wavelengths, species, delivery routes, and endpoints; mitochondrial, vascular, glial, lymphatic, network-mediated, and systemic pathways are therefore presented as plausible mechanisms at different evidence levels, not as an established causal chain. These constraints limit causal inference while leaving the central hypotheses directly testable.

## 11 Conclusion

The history of dtPBM is neither a simple sequence of successes nor a simple sequence of failures. Near-infrared light can traverse the human scalp and skull, and transcranial illumination can alter measurable human brain physiology. Clinical efficacy, however, has been inconsistent, and the variables that determine biological dose remain incompletely resolved.

NEST showed that one fixed 808-nm, single-session whole-vault regimen did not improve outcome in a definitive acute-stroke trial. The forehead pivot improved optical access and target reproducibility. ELATED exemplified a shift to lower irradiance with broadly similar radiant exposure, without isolating the clinical effect of that change. R61 then showed that changing optical parameters can change the sign of an acute physiological response.

Three experiments now have disproportionate value: measure or credibly estimate target exposure in individual participants and link it to an independent engagement measure; separate peak irradiance, average irradiance, duty cycle, frequency, and duration rather than treating J/cm^2^ as sufficient dose; and compare matched optical regimens at different cortical montages to determine whether the physiological response reorganizes with network connectivity. These experiments are most informative in indications where an accessible target and a disease-relevant biomarker or circuit can be specified prospectively. When their results would change treatment design, they should precede further scale-up.

Before making trials larger, the field should establish what dose reaches the relevant tissue, what biological response it produces, where that response begins, and whether the measured engagement is relevant to the disease being treated.

## Data Availability

Published data discussed in this article are available through the cited sources. Aggregate results from the unpublished NEST-2 depth-stratified reanalysis are reported in the text. Individual participant-level NEST-2 data are not publicly available. The original sponsor, PhotoThera Inc., was dissolved; access to surviving individual-level data is subject to current custody and applicable contractual restrictions. Requests for the derived depth-classification dataset and analysis code underlying the NEST-2 reanalysis may be directed to the corresponding author and will be considered within those constraints.
